# Differences in the characteristics and contemporary cardiac outcomes of patients with light-chain versus transthyretin cardiac amyloidosis

**DOI:** 10.1371/journal.pone.0255487

**Published:** 2021-08-09

**Authors:** Osnat Itzhaki Ben Zadok, Mordehay Vaturi, Iuliana Vaxman, Zaza Iakobishvili, Noa Rhurman-Shahar, Ran Kornowski, Ashraf Hamdan

**Affiliations:** 1 Department of Cardiology, Rabin Medical Center, Petah Tikva, Israel; 2 Sackler Faculty of Medicine, Tel Aviv University, Tel Aviv, Israel; 3 Davidoff Cancer Center, Institute of Hematology, Rabin Medical Center, Petah- Tikva, Israel; 4 Clalit” Health Services, Tel-Aviv District, Israel; 5 Raphael Recanati Genetic Institute, Rabin Medical Center, Petah Tikva, Israel; Scuola Superiore Sant’Anna, ITALY

## Abstract

**Aims:**

To compare the baseline cardiovascular characteristics of immunoglobulin light-chain (AL) and amyloid transthyretin (ATTR) cardiac amyloidosis (CA) and to investigate patients’ contemporary cardiac outcomes.

**Methods:**

Single-center analysis of clinical, laboratory, echocardiographic and cardiac magnetic resonance imaging (CMRi) characteristics of AL and ATTR-CA patients’ cohort (years 2013–2020).

**Results:**

Included were 67 CA patients of whom 31 (46%) had AL-CA and 36 (54%) had ATTR-CA. Patients with ATTR-CA versus AL-CA were older (80 (IQR 70, 85) years versus 65 (IQR 60, 71) years, respectively, p<0.001) with male predominance (p = 0.038). Co-morbidities in ATTR-CA patients more frequently included diabetes mellitus (19% versus 3.0%, respectively, p = 0.060) and coronary artery disease (39% versus 10%, respectively, p = 0.010). By echocardiography, patients with ATTR-CA versus AL-CA had a trend to worse left ventricular (LV) ejection function (50 (IQR 40, 55)% versus 60 (IQR 45, 60)%, respectively, p = 0.051), yet comparable LV diastolic function. By CMRi, left atrial area (31 (IQR 27, 36)cm^2^ vs. 27 (IQR 23, 30)cm^2^, respectively, p = 0.015) and LV mass index (109 (IQR 96, 130)grams/m^2^ vs. 82 (IQR 72, 98)grams/m^2^, respectively, p = 0.011) were increased in patients with ATTR-CA versus AL-CA. Nevertheless, during follow-up (median 20 (IQR 10, 38) months), patients with AL-CA were more frequently admitted with heart failure exacerbations (HR 2.87 (95% CI 1.42, 5.81), p = 0.003) and demonstrated increased mortality (HR 2.51 (95%CI 1.19, 5.28), p = 0.015).

**Conclusion:**

Despite the various similarities of AL-CA and ATTR-CA, these diseases have distinct baseline cardiovascular profiles and different heart failure course, thus merit tailored-cardiac management.

## Introduction

Cardiac amyloidosis (CA) is most commonly caused by the extracellular deposition of insoluble amyloid fibrils of either immunoglobulin light chain (AL) or amyloid transthyretin (ATTR) [1], and is considered to portend a poor prognosis [2]. This distinctive cardiomyopathy is most frequently categorized as heart failure with preserved ejection fraction (HFpEF), and patients with CA are oftentimes described with advanced heart failure (HF) and significant volume overload [3]. Electrical conduction abnormalities as well as atrial and ventricular arrhythmias are other common findings in both AL-CA and ATTR-CA.

The shared clinical and imaging characteristics of AL-CA and ATTR-CA, which most frequently present at advanced or end-stage amyloid heart disease, have often resulted in the combination of both diseases under one umbrella. However, important differences do exist, and most probably derivate from their specific etiology and pathophysiology [4, 5]. Defining these differences may pave the way for an earlier, targeted diagnosis of AL or ATTR CA and improve our understanding of disease progression and management.

We sought to compare in a contemporary cohort of patients with AL-CA and ATTR-CA their clinical, laboratory and imaging characteristics, and to investigate patients’ long-term survival and HF-related complications.

## Methods

The study population was comprised of consecutive AL-CA and ATTR-CA patients treated at a tertiary institution (Rabin Medical Center, Israel) between the years 2013–2020. For all patients, electronic medical records and echocardiographic and cardiac magnetic resonance (CMR) examinations were retrospectively reviewed.

The diagnosis criteria of ATTR-CA and AL-CA has been previously described [4]. Cardiac ATTR was defined as the combination of symptoms with an echocardiogram consistent with or suggestive of cardiac amyloidosis, grade 2 or 3 cardiac uptake on 99mTc-DPD scintigraphy in the absence of a monoclonal gammopathy [6]. In the presence of a monoclonal gammopathy, a cardiac biopsy positive for ATTR was warranted. Following a histological or non-invasive diagnosis of ATTR, all patients were referred to TTR genetic testing to differentiate between mutant ATTR and wild-type ATTR. The diagnosis and staging of AL amyloidosis were according to consensus criteria and required to prove the presence of amyloid deposition in tissue biopsy by Congo red staining [7, 8]. Further protein analysis confirming light chain deposits was made by immunohistochemistry [9]. Mass spectrometry proteomic analysis was undertaken in selected cases. The diagnosis of cardiac amyloid involvement in AL was based on either CMR imaging (with evidence of congo-red tissue staining elsewhere) or endomyocardial biopsy (EMB). Patients who were deferred by their treating physician from CMR or EMB (high-risk patients) were provisionally diagnosed based on typical echocardiographic features (concentric LV thickening and diastolic dysfunction), as previously reported [10]. Patients were excluded if the diagnosis of CA did not meet the above criteria.

On echocardiography, the left atrial and ventricular diameters and left ventricular (LV) ejection fraction (LVEF) were measured according to accepted guidelines [11]. Relative wall thickness (RWT) was calculated as 2 times LV posterior wall (PW) diastolic thickness divided by LV diastolic diameter [11]. LV mass was calculated according to the Devereux formula [12]: 1.04 ((LV diastolic diameter + interventricular septal (IVS) diameter + LV PW diastolic thickness)^3^- (LV diastolic diameter^3^)-13.6. Right ventricular (RV) function was evaluated qualitatively by visual assessment [13]. The pulmonary artery systolic pressure was estimated from the peak velocity of the tricuspid regurgitation jet and estimated right atrial pressure based on inferior vena cava diameter and distensibility [13]. LV diastolic function was assessed by integrating mitral flow pattern, tissue Doppler imaging, indexed left atrial volume, and systolic pulmonary pressure [14]. Cut-off values for defining abnormalities in the reported echocardiographic variables were chosen according to published reference guidelines in the general population [11, 15–18].

A sub-group of the cohort underwent CMR with a 1.5 Tesla scanner. The following standardized protocol was applied: contiguous cine short axis views covering the whole LV; 3 cine long axis views of the LV (two- three and four-chamber) planned on the short-axis orientation; for delayed enhancement contiguous short axis views covering the whole LV and 3 long axis views of the LV (two- three and four-chamber) were acquired. LV end-diastolic wall thickness of the septum and lateral wall were measured in a basal short-axis view. For volume measurements, endocardial borders were traced manually at end diastole and end systole. LV end-diastolic volume (LVEDV) and LV end-systolic volume (LVESV) were assessed and indexed to body mass index. LVEF was calculated by using Simpson’s rule.

The primary endpoint of this study was overall survival, and the secondary endpoint was survival free of the occurrence of malignant ventricular arrhythmias (defined as sustained ventricular tachycardia or ventricular fibrillation) or high degree atrioventricular block. Other clinical endpoints were HF-related admissions or arrhythmias-related admissions. Only unplanned admissions post-diagnosis were reported. Chronic kidney disease was defined by estimated glomerular filtration rate (eGFR)<60 ml/min as calculated by the CKD-EPI formula. Mortality during follow-up was determined for all patients through the Israeli National Population Registry. The study protocol was approved by the Rabin Medical Center Institutional Review Board.

The statistical analysis was carried out using SAS Statistical Software, Version 9.4 (SAS Institute Inc., Cary, NC). Continuous variables were presented by median and interquartile 25^th^, 75^th^ range. Categorical variables were presented by (N, %). T-Test was used to compare the values of continuous variables, displaying normal distribution between study groups and the Wilcoxon test was used for non-Gaussian distributions. Chi-square was used to compare the values of categorical variables, displaying normal distribution and the Fisher’s exact test was used for non-Gaussian distributions. Overall survival was defined as the time from diagnosis to death from any cause. For time to death and for the combined endpoint of malignant ventricular arrhythmias/high degree atrioventricular block or death, the survival curve during study follow-up was assessed by Kaplan-Meier survival analysis, with the log-rank test. The Cox proportional hazards model was used to calculate hazard ratios (HR) and for multivariable analysis.

For analysis of survival endpoints which did not include death (time to subsequent HF exacerbations or arrhythmias-related admissions) death with no admission was considered as a competing risk. The Anderson Gill method, in the Cox model, was used to analyze repeated admissions.

Two-sided p values less than 0.05 were considered statistically significant.

## Results

### Baseline clinical parameters

The study cohort included 67 patients with a diagnosis of CA of whom 31 (46%) and 36 (54%) patients were diagnosed with AL and ATTR CA, respectively. Seven patients with ATTR were diagnosed with mutant ATTR. Patients’ baseline characteristics and amyloid type-specific parameters are presented in **Table 1** and **S1** and **S2 Tables**. Patients with ATTR-CA were significantly older than patients with AL-CA (median age at the diagnosis of ATTR was 80 (IQR 70, 85) years vs. 65 (IQR 60, 71) years in AL, p<0.001) with male predominance (78% vs. 52% p = 0.038). Moreover, patients with ATTR versus AL-CA more frequently presented with cardiovascular co-morbidities including diabetes mellitus (19% vs. 3%, respectively, p = 0.060), coronary artery disease (39% vs. 10%, respectively, p = 0.010) and acute myocardial infarction (19% vs. 0%, respectively, p = 0.011). The rate of chronic kidney disease was similar between groups (36% of study patients, p = 0.615). The prevalence of prior carpal tunnel syndrome was higher in patients with ATTR-CA versus AL-CA (56% vs. 29%, respectively, p = 0.027). Laboratory tests including baseline levels of NT-proBNP and troponin were comparable between patients with AL-CA and ATTR-CA. Ten patients diagnosed with AL-CA (32%) were treated with anti-CD38 monoclonal antibodies as 2^nd^ or 3^rd^ line treatment.

**Table 1:** Baseline characteristics of patients with cardiac amyloidosis stratified by the misfolded amyloid protein.

	AL (n = 31)	ATTR (n = 36)	p-value
Age at amyloidosis diagnosis (years)	65 (60, 71)	80 (70, 85)	<0.001
Sex, male (%)	16 (52)	28 (78)	0.038
Body mass index (Kg/m^2^)	28 (25, 35)	25 (23, 29)	0.141
Diabetes mellitus (%)	1 (3)	7 (19)	0.060
Hypothyroidism (%)	2 (6)	3 (8)	1.000
Hypertension (%)	10 (32)	17 (47)	0.318
Dyslipidemia (%)	9 (29)	15 (42)	0.314
Family history of ischemic heart disease (%)	6 (19)	2 (6)	0.132
Coronary artery disease (%)	3 (10)	14 (39)	0.010
Myocardial infarction (%)	0 (0)	7 (19)	0.011
Atrial fibrillation (%)	7 (23)	16 (44)	0.075
Moderate-severe aortic stenosis (%)	0 (0)	1 (3)	1.000
Carpal tunnel syndrome (%)	9 (29)	20 (56)	0.027
Past smoker (%)	3 (10)	9 (25)	0.117
Alcohol consumption (%)	0 (0)	0 (0)	
NYHA FC (%)			0.130
1	8 (26)	11 (31)	
2	9 (29)	18 (50)	
3	13 (42)	7 (19)	
4	1 (3)	0 (0)	
**Laboratory parameters**			
Hemoglobin (g/dL)	13 (12, 14)	13 (12, 14)	0.642
Creatinine (mg/dl)	1.1 (0.77, 1.3)	1.0 (0.86, 1.5)	0.984
Estimated GFR by CKD-EPI* (ml/min/1.73m^2^)	70 (53, 83)	68 (46, 83)	0.615
Sodium (mEq/L)	139 (138, 142)	139 (136, 141)	0.582
Potassium (mEq/L)	4.3 (3.9, 4.7)	4.5 (4.3, 4.8)	0.091
Albumin (g/dL)	3.9 (3.5, 4.3)	3.9 (3.8, 4.2)	0.690
AST (U/L)	27 (20, 35)	26 (21, 34)	0.711
*γ*GT (U/L)	52 (26, 102)	46 (28, 104)	0.740
NT-proBNP (pg/ml)^	3370 (1661, 13924)	3467 (1202, 6192)	0.598
Troponin T (ng/L)^^	84 (52, 134)	62 (49, 99)	0.290
ECG Holter monitoring performed at diagnosis	14 (45)	24 (67)	0.084
**Medications (%)**			
Mineralocorticoid-receptor antagonists	11 (35)	19 (53)	0.141
Furosemide	26 (84)	30 (83)	0.745
Beta-blockers	9 (29)	15 (42)	0.442
Digoxin	1 (3)	2 (6)	1.000
Renin-angiotensin-aldosterone system inhibitors	11 (35)	9 (25)	0.421
Anti-arrhythmic drugs	2 (6)	4 (11)	0.681
Oral anti-coagulation therapy	2 (6)	20 (55)	<0.001

Data are presented as medians (25^th^, 75^th^ quartiles) or as percentages, as appropriate.

**Abbreviations**: AL, immunoglobulin light-chain; AST, aspartate aminotransferase; ATTR, amyloid-transthyretin; CA, cardiac amyloidosis; GFR, glomerular filtration rate; *γ*GT, gamma-glutamyltransferase; NYHA FC, New-York Heart Association functional class; NT-proBNP, N-terminal-pro hormone brain natriuretic peptide.

*CKD-EPI; Chronic Kidney Disease Epidemiology Collaboration

^ NT-proBNP levels at baseline were available in 22 (71%) and 26 (72%) of AL and ATTR-CA patients, respectively.

^ ^ Troponin T normal laboratory range <13ng/L.

### Baseline echocardiographic evaluation

Patients’ baseline echocardiographic findings are presented in **Table 2**. The median time from the reported echocardiography evaluation to the diagnosis of CA was -5 (IQR -40, +30) days, regardless of amyloid type. Patients with ATTR-CA versus AL-CA had a trend towards worse LV systolic function as demonstrated by reduced LVEF (50 (IQR 40, 55)% vs. 60 (IQR 45, 60)%, respectively, p = 0.051), yet comparable LV diastolic function. Patients with ATTR-CA vs. AL-CA presented with thicker septal (1.6 (IQR 1.5, 1.9) cm vs. 1.40 (IQR 1.3, 1.6) cm, respectively, p = 0.004) and posterior (1.5 (IQR 1.3, 1.8) cm vs. 1.30 (IQR 1.2, 1.5) cm, respectively, p = 0.017) LV walls. Moreover, LV mass was significantly increased in patients with ATTR versus AL-CA even after adjusting to body surface area (148 (IQR 129, 198) grams/m^2^ vs. 113 (IQR 97, 138) grams/m^2^, respectively, p = 0.027). Notably, rates of arterial hypertension and clinically significant aortic stenosis were similar between groups. Similar quantitative results were observed in a subgroup analysis that included only AL-CA vs. ATTR-CA male patients (septal thickness 1.5 (IQR 1.3, 1.7) cm vs. 1.6 (IQR 1.5, 1.9) cm, respectively, p = 0.012 and LV mass index (115 (IQR 96, 146) grams/m^2^ vs. 160 (IQR 138, 203) grams/m^2^, respectively, p = 0.016).

**Table 2:** Echocardiographic and cardiac magnetic resonance imaging findings among patients with CA stratified by amyloid subtype.

Echocardiography studies	AL (n = 31)	TTR (n = 36)	p-value
LVEF (median)	60 (45, 60)	50 (40, 55)	0.051
RV dysfunction (%)	11 (35)	15 (42)	0.606
Posterior wall (cm)	1.30 (1.20, 1.50)	1.50 (1.30, 1.80)	0.017
Intraventricular septum (cm)	1.40 (1.30, 1.60)	1.60 (1.45, 1.90)	0.004
Wall thickness ≥1.4cm (%)	20 (65)	31 (86)	0.048
LVEDD (cm)	4.1 (3.9, 4.5)	4.3 (3.8, 4.6)	0.645
LVESD (cm)	2.8 (2.5, 3.3)	3.1 (2.6, 3.5)	0.092
Relative wall thickness	0.65 (0.57, 0.81)	0.74 (0.60, 0.92)	0.111
LA area (cm^2^)	24 (20, 26)	27 (24, 31)	0.005
LA diameter (cm)	4.3 (3.7, 4.6)	4.4 (4.0, 4.7)	0.202
LV mass (grams)	212 (168, 280)	253 (220, 330)	0.042
LV mass index (grams/m^2^)	113 (97, 138)	148 (129, 198)	0.027
TAPSE (mm)	15 (11, 18)	14 (12, 17)	0.928
Diastolic grade 2, 3 (%)	26 (84)	20 (56)	0.306
E/A	2.1 (1.6, 3.0)	2.8 (1.6, 3.3)	0.521
Deceleration time (msec)	144 (117, 193)	134 (118, 148)	0.476
e’ lateral	4.0 (4.0, 5.0)	5.0 (3.9, 6.0)	0.522
E/e’	20 (17, 26)	17 (14, 20)	0.103
SPAP (mmHg)	41 (31, 51)	42 (35, 55)	0.768
**CMR studies**	n = 22	n = 24	
LA area (cm^2^)	27 (23, 30)	31 (27, 36)	0.015
LVEF (median), %	54 (51, 67)	52 (39, 62)	0.206
Stroke index (ml/m^2^)	29 (26, 40)	36 (30, 44)	0.118
LVEDVi (ml/m^2^)	63 (53, 76)	81 (59, 94)	0.038
LVESVi (ml/m^2^)	26 (20, 33)	43 (22, 60)	0.167
Cardiac output (L/min)	4.4 (4.1, 5.4)	5.6 (4.8, 6.8)	0.065
Septal thickness (cm)	1.6 (1.3, 1.8)	1.9 (1.5, 2.1)	0.040
LV mass (grams)	150 (120, 180)	211 (149, 264)	0.022
LV mass index (grams/m^2^)	82 (72, 98)	109 (96, 130)	0.011
RVEF (median), %	43 (40, 51)	45 (35, 62)	0.709

Data are presented as medians (25^th^, 75^th^ quartiles) or as percentages, as appropriate.

**Abbreviations**: AL, immunoglobulin light-chain; ATTR, amyloid-transthyretin; CA, cardiac amyloidosis; LA, left atria, LV, left ventricular; LVEDD, left ventricular end diastolic diameter; LVESD, left ventricular end systolic diameter; LVEDVi, left ventricular end diastolic volume index; LVESVi, left ventricular end systolic volume index; LVEF, left ventricular ejection fraction; RV, right ventricular; RVEF, right ventricular ejection fraction RWT, relative wall thickness; SPAP, systolic pulmonary artery pressure; TAPSE, tricuspid annular plane systolic excursion.

### Baseline cardiac magnetic resonance imaging

Twenty-two (71%) AL-CA patients and 24 (67%) ATTR-CA patients completed CMR examination as part of their baseline evaluation (**Table 2**). LV and RV systolic function as demonstrated by CMR were comparable in AL-CA and ATTR-CA patients. However, LA area (31 (IQR 27, 36) cm^2^ vs. 27 (IQR 23, 30) cm^2^, respectively, p = 0.015), LV septal thickness (1.9 (IQR 1.5, 2.1) cm vs. 1.6 (IQR 1.3, 1.8) cm, respectively, p = 0.040) and LV mass index (109 (IQR 96, 130) grams/m^2^ vs. 82 (IQR 72, 98) grams/m^2^, respectively, p = 0.011) were significantly increased in patients with ATTR-CA versus AL-CA. The majority of CA patients (n = 38) demonstrated subendocardial late gadolinium enhancement pattern, while 8 patients (5 AL-CA and 3 ATTR-CA) demonstrated a transmural late gadolinium enhancement pattern.

### Survival and cardiac-related admissions

**Fig 1:**
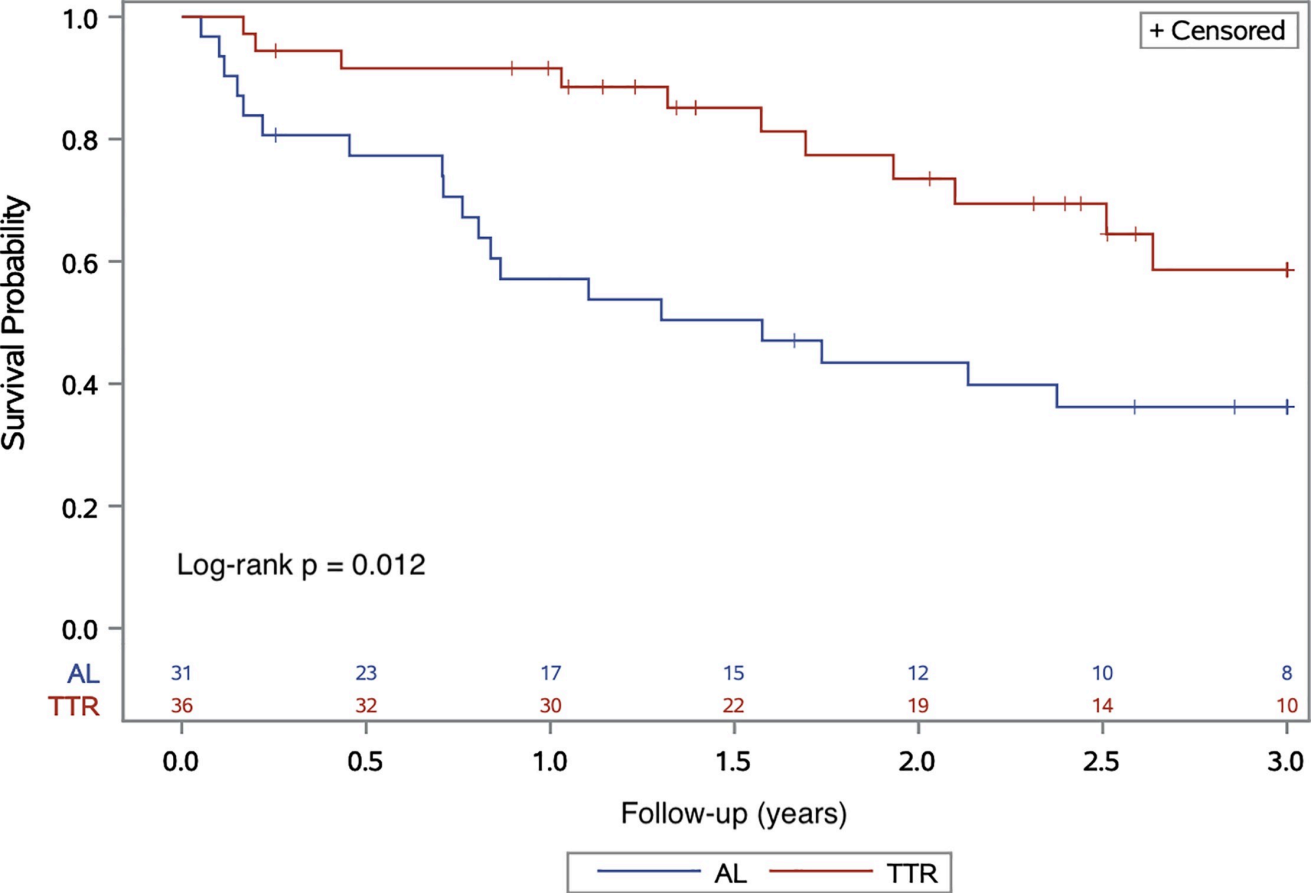
Kaplan-Meier curve of all-cause survival of patients with CA stratified by the pathogenetic amyloid subtype. Abbreviations: AL, immunoglobulin light-chain; ATTR, amyloid-transthyretin, CA, cardiac amyloidosis.

Over study observational follow-up (median 20 (IQR 10, 38) months), all-cause mortality in patients with AL-CA versus ATTR-CA was significantly higher (HR 2.51 (95%CI 1.19, 5.28), p = 0.015) (**Fig 1**). When analyzed for ATTR sub-populations, survival was similar between mutant ATTR-CA versus wild-type ATTR-CA (Log-rank p = 0.560), yet higher when compared with AL-CA (AL vs. mutant ATTR-CA, Log-rank p = 0.044; AL vs. wild-type, Log-rank p = 0.012, **S1 Fig**). The cause of death was cardiac in 23% (n = 7) vs. 11% (n = 4) in patients with AL-CA versus ATTR-CA, respectively. Similar quantitative results were observed in a subgroup analysis that included only AL-CA patients with <20% plasma cells at bone marrow biopsy (HR 2.50 (95%CI 1.03, 6.04), p = 0.042). AL-CA versus ATTR-CA was found as an independent predictor for mortality on a multivariable analysis adjusted to age, sex and renal function (HR 2.93 (95%CI 1.1, 7.6), p = 0.027), **Table 3**). The composite endpoint of malignant ventricular arrhythmias/high degree atrioventricular block or death was higher in patients with AL-CA versus ATTR-CA at 1-year follow-up (HR 4.45 (95%CI 1.45, 13.6), p = 0.009), yet comparable at 3-year (Log-rank p = 0.208) (**S2 Fig**).

**Table 3:** Multivariate analysis of predictors for death in patients with CA.

Parameter	HR (95% CI)	p-value
**AL-CA versus ATTR-CA**	**2.93 (1.1, 7.6)**	**0.027**
Age at diagnosis	1.01 (0.97, 1.06)	0.549
Female sex	1.20 (0.55, 2.6)	0.642
Estimated GFR by CKD-EPI*	0.99 (0.98, 1.01)	0.606

**Abbreviations**: AL, immunoglobulin light-chain; ATTR, amyloid-transthyretin; CA, cardiac amyloidosis; CI, confidence interval; GFR, glomerular filtration rate; HR, hazard ratio.

*CKD-EPI; Chronic Kidney Disease Epidemiology Collaboration.

We also used the Anderson Gil Model to investigate the recurrence of HF-related admissions controlling for death as a competing risk. During follow-up, patients with AL-CA versus ATTR-CA were more frequently admitted with HF exacerbations (HR 2.87 (95% CI 1.42, 5.81), p = 0.003). No differences were noted in the frequency of arrhythmias-related admissions (p = 0.890).

## Discussion

This study which included a contemporary cohort of patients with cardiac amyloid involvement highlights the distinct clinical and prognostic cardiac profiles of patients with AL-CA versus ATTR-CA. We found that although patients with AL-CA were younger, with fewer cardiovascular comorbidities and more benign echocardiographic phenotype, they had increased rates of HF exacerbations and all-cause mortality compared to patients with ATTR-CA.

Several studies have sought to investigate the association between the different CA subtypes and amyloid cardiac involvement. Rapezzi et al. have observed increased LV wall thickness and LV mass among men in a cohort of patients with ATTR-CA (mainly mutant) versus AL-CA, as assessed by echocardiography [19]. In a CMR-based study, Martinez-Naharro et al. have demonstrated higher LV and RV mass index as well as an increase in myocardial extracellular volume in patients with ATTR-CA compared to patients with AL-CA [20]. Our observations are in line with these findings and further characterize each CA sub-population from a clinical cardiac perspective. Patients with ATTR-CA versus AL-CA were older, had increased rates of diabetes mellitus, coronary artery disease and myocardial infarction as well as impaired LV systolic function by echocardiography. Moreover, the composite endpoint of malignant arrhythmias and death at 1-year was higher in patients with AL-CA versus ATTR-CA. At longer follow-up, the relative rate of malignant arrhythmias increased in ATTR-CA versus AL-CA patients with no significant differences between study groups, most probably due to survival bias. In summary, patients with ATTR-CA revealed a “sicker” baseline cardiovascular profile at the time of CA diagnosis. Nevertheless, as shown, patients with ATTR-CA (either wild-type or mutant) had better overall and HF-related prognosis compared to patients with AL-CA, findings which persisted even after the exclusion of patients with increased percentage of bone marrow plasma cells, an adverse prognostic factor in AL-CA [21]. To note, we observed similar levels of NT-proBNP between patients with AL-CA and ATTR-CA. However, considering the older age, increased LV mass and worse LV systolic function of ATTR-CA patients, it is plausible to infer that patients with AL-CA had relatively higher NT-proBNP levels, again signifying for worse HF status [22].

Both AL-CA and ATTR-CA are associated with myocardial extracellular fibril deposition, which ultimately results in myocardial restriction and diastolic dysfunction [5]. Nevertheless, over 2 decades ago, Dubrey et al. have postulated for a unique toxic component of AL-CA in addition to its recognized infiltrative pathophysiology [23], a hypothesis that was later confirmed in an isolated mouse heart model [24]. Moreover, recently, a proteomics analysis by Kourelis et al. characterized ATTR by a higher abundance of complement and contractile proteins and AL by a higher abundance of keratins, suggesting different mechanisms of tissue damage [25]. Importantly, other than the toxicity of the immunoglobulin light chains, the systemic, particularly renal, involvement in AL-CA adversely contributes to patients’ morbidity and mortality [26]. We believe that our contemporary observations, documented in an era of advanced AL-CA suppressive-therapies, support those prior reports by demonstrating an inverse association between the better cardiovascular phenotype of AL-CA patients and their worse HF status and overall survival. Moreover, our findings highlight the discrepancy between the well-documented prognostic imaging parameters in non-amyloid cardiomyopathy and the distinct pathophysiology of AL cardiomyopathy.

## Limitations

This study has several limitations. First, our study is limited by its relatively small sample size and single-center nature possibly limiting generalizability. Moreover, the rate of patients with mutant ATTR versus wild-type ATTR in this cohort was low, and thus our observations may not accurately reflect the echocardiographic findings in this sub-population. Second, although global longitudinal strain analysis is an important element in the echocardiographic evaluation of cardiac amyloid involvement, these data were missing in the majority of our study patients, and thus not presented. This is because global longitudinal strain analysis was not routinely used at our institution during most of the study long-term observation period.

In conclusion, despite the shared similarities of AL-CA and ATTR-CA, these diseases have distinct baseline cardiovascular profiles and different HF clinical course. We believe our findings will help promote a differential diagnostic work-up, which is amyloid-subtype directed. The pathophysiology and management of light-chain cardiac toxicity merit further study in order to improve HF-related prognosis of AL-CA patients.

## Supporting information

S1 Fig Kaplan-Meier curve of all-cause survival of patients with CA stratified by the pathogenetic amyloid subtype: (**A**) mutant ATTR-CA versus wild-type ATTR-CA and (**B**) mutant and wild-type ATTR-CA versus AL-CA. Abbreviations: AL, immunoglobulin light-chain; ATTR, amyloid-transthyretin, CA, cardiac amyloidosis.
(DOCX)

S2 Fig Kaplan-Meier curve of the combined endpoint of survival free of malignant arrhythmias of patients with CA stratified by the pathogenetic amyloid subtype at 1-year (**A**) and 3-year (**B**) follow-up. Abbreviations: AL, immunoglobulin light-chain; ATTR, amyloid-transthyretin, CA, cardiac amyloidosis.
(DOCX)

S1 Table Baseline characteristics of patients with AL cardiac amyloidosis. Data are presented as medians (25^th^, 75^th^ quartiles) or as percentages, as appropriate. ^ Kumar S, Dispenzieri A, Lacy MQ, Hayman SR, Buadi FK, Colby C, et al. Revised prognostic staging system for light chain amyloidosis incorporating cardiac biomarkers and serum free light chain measurements. Journal of clinical oncology 2012;30(9):989–95. Nine (29%) patients had missing NT-proBNP levels at baseline, thus precluding the calculation of cardiac prognostic stage. ^#^ Palladini G, Hegenbart U, Milani P, Kimmich C, Foli A, Ho AD, et al. A staging system for renal outcome and early markers of renal response to chemotherapy in AL amyloidosis. Blood. 2014;124(15):2325–32. Eight (26%) patients had missing urine-24 hour protein assessment at baseline, thus precluding the calculation of renal stage. Abbreviations: AL, immunoglobulin light-chain.
(DOCX)

S2 Table Baseline characteristics of patients with ATTR cardiac amyloidosis. Data are presented as percentages, as appropriate. Abbreviations: ATTR, transthyretin amyloidosis.
(DOCX)
