# Supplementary figures and images for: Differences in the characteristics and contemporary cardiac outcomes of patients with light-chain versus transthyretin cardiac amyloidosis
Source: PLoS One. 2021 Aug 9;16(8):e0255487. doi: 10.1371/journal.pone.0255487 (PMC8351987; doi:10.1371/journal.pone.0255487)

**Supplementary Figure 1:**


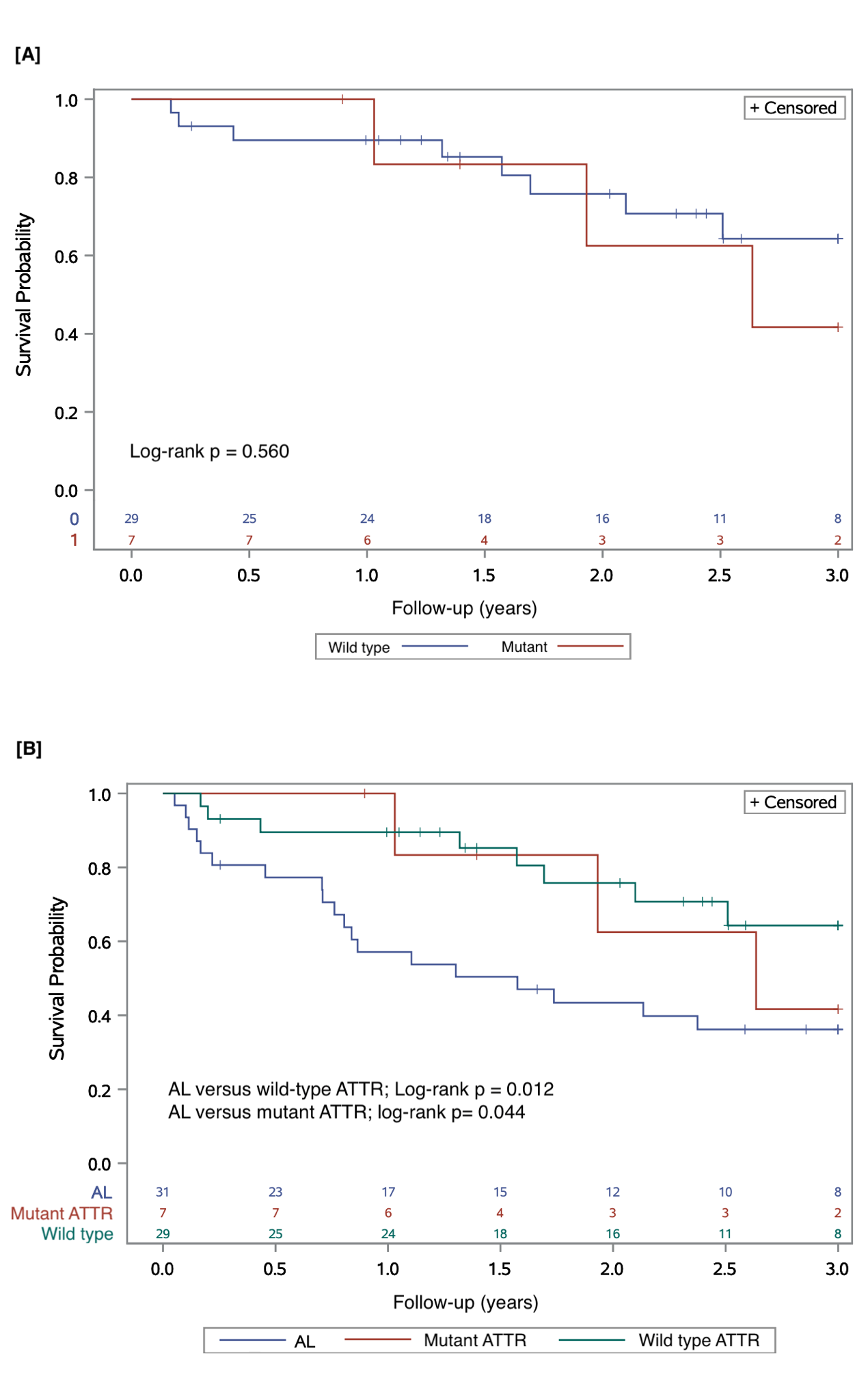

Supplement: S1 Fig — Kaplan-Meier curve of all-cause survival of patients with CA stratified by the pathogenetic amyloid subtype: (A) mutant ATTR-CA versus wild-type ATTR-CA and (B) mutant and wild-type ATTR-CA versus AL-CA. Abbreviations: AL, immunoglobulin light-chain; ATTR, amyloid-transthyretin, CA, cardiac amyloidosis. (DOCX) [file pone.0255487.s001.docx]

**Supplementary Figure 2:**


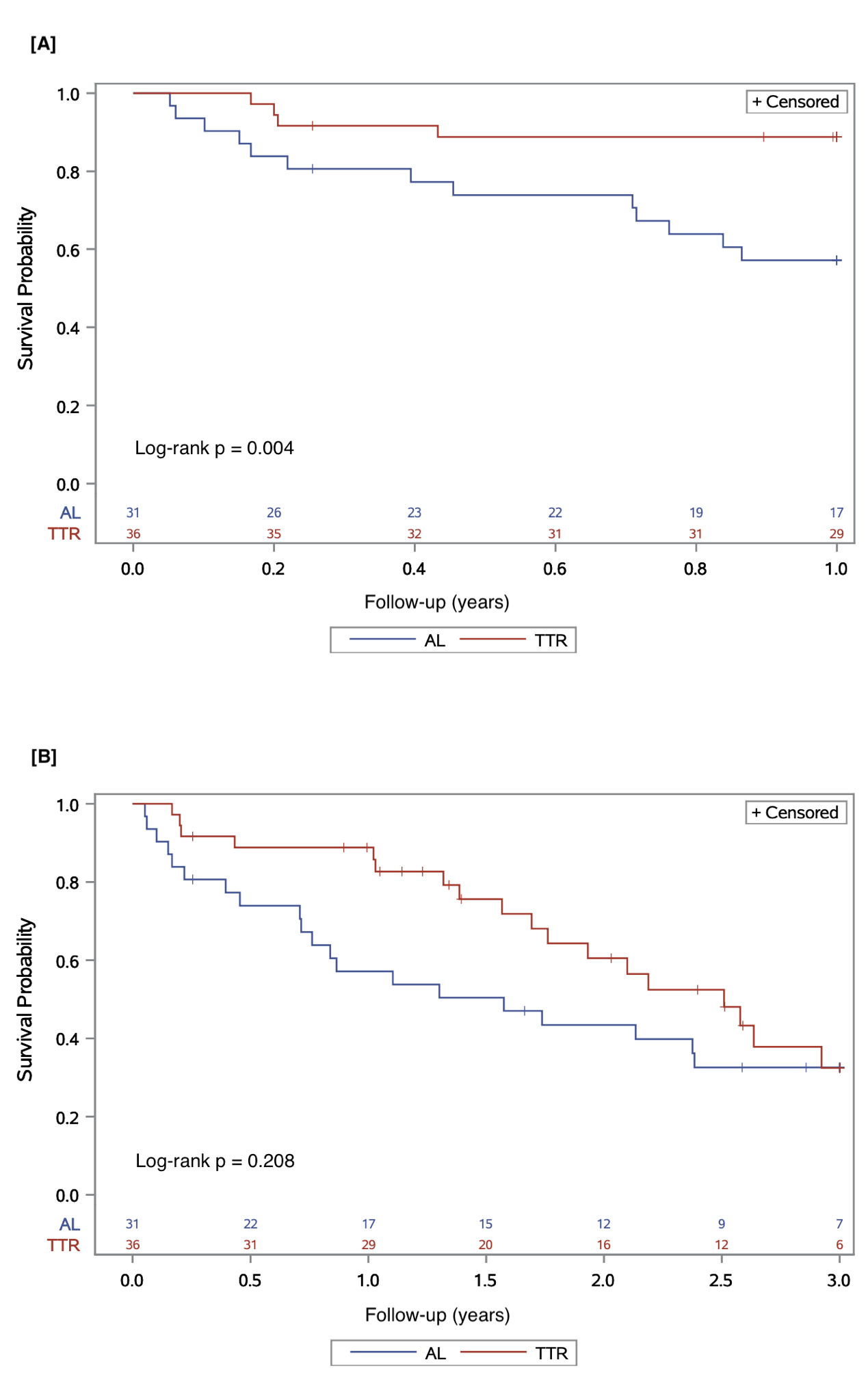

Supplement: S2 Fig — Kaplan-Meier curve of the combined endpoint of survival free of malignant arrhythmias of patients with CA stratified by the pathogenetic amyloid subtype at 1-year (A) and 3-year (B) follow-up. Abbreviations: AL, immunoglobulin light-chain; ATTR, amyloid-transthyretin, CA, cardiac amyloidosis. (DOCX) [file pone.0255487.s002.docx]
