# Supplementary material for: Differences in the characteristics and contemporary cardiac outcomes of patients with light-chain versus transthyretin cardiac amyloidosis
Source: PLoS One. 2021 Aug 9;16(8):e0255487. doi: 10.1371/journal.pone.0255487 (PMC8351987; doi:10.1371/journal.pone.0255487)
Supplement: S1 Table — Data are presented as medians (25th, 75th quartiles) or as percentages, as appropriate. ^ Kumar S, Dispenzieri A, Lacy MQ, Hayman SR, Buadi FK, Colby C, et al. Revised prognostic staging system for light chain amyloidosis incorporating cardiac biomarkers and serum free light chain measurements. Journal of clinical oncology 2012;30(9):989–95. Nine (29%) patients had missing NT-proBNP levels at baseline, thus precluding the calculation of cardiac prognostic stage. # Palladini G, Hegenbart U, Milani P, Kimmich C, Foli A, Ho AD, et al. A staging system for renal outcome and early markers of renal response to chemotherapy in AL amyloidosis. Blood. 2014;124(15):2325–32. Eight (26%) patients had missing urine-24 hour protein assessment at baseline, thus precluding the calculation of renal stage. Abbreviations: AL, immunoglobulin light-chain. (DOCX) [file pone.0255487.s003.docx]

**Supplementary Table 1**

|  | **AL** (n=31) |
| --- | --- |
| **Revised prognostic staging system^** |  |
| Stage I | 0 (0) |
| Stage II | 4 (18) |
| Stage III | 6 (27) |
| Stage IV | 12 (55) |
| **Renal staging system**^#^ |  |
| Stage I | 18 (78) |
| Stage II | 5 (22) |
| Stage III | 0 (0) |
| **Amyloid systemic involvement (%)** |  |
| **Cardiac** | 31 (100) |
| *Cardiac magnetic resonance imaging* | 26 (84) |
| *Endomyocardial biopsy* | 4 (13) |
| *Echocardiography-based diagnosis* | 4 (13) |
| Bone marrow | 15 (48) |
| Fat pad | 6 (19) |
| Tongue | 2 (6) |
| Gastrointestinal | 4 (13) |
| Mass spectrometry proteomic analysis | 5 (16) |
| **Laboratory parameters** |  |
| Kappa-light chain | 2.9 (1.0, 27) |
| Lambda-light chain | 24 (8, 63) |
| Light chain difference | 43 (23, 73) |
| Blood marrow plasma cells (%) | 20 (12, 40) |
| Urine 24-protein (mg) | 518 (192, 1145) |
| **AL Targeted therapy** |  |
| Bortezomib | 28 (90) |
| Lenalidomide | 15 (48) |
| Pomalidomide/thalidomide | 6 (19) |
| Daratumumab | 10 (32) |
| Autologous stem cell transplantation | 1 (3) |
| Not-treated | 2 (6) |
