# Supplementary material for: Differences in the characteristics and contemporary cardiac outcomes of patients with light-chain versus transthyretin cardiac amyloidosis
Source: PLoS One. 2021 Aug 9;16(8):e0255487. doi: 10.1371/journal.pone.0255487 (PMC8351987; doi:10.1371/journal.pone.0255487)
Supplement: S2 Table — Data are presented as percentages, as appropriate. Abbreviations: ATTR, transthyretin amyloidosis. (DOCX) [file pone.0255487.s004.docx]

**Supplementary Table 2:**

|  | **ATTR** (n=36) |
| --- | --- |
| **Amyloid systemic involvement (%)** |  |
| **Cardiac** | 35 (100) |
| *Cardiac magnetic resonance imaging* | 27 (75) |
| *Endomyocardial biopsy* | 2 (6) |
| *Technetium-Pyrophosphate Scan* | 28 (80) |
| Mass spectrometry proteomic analysis | 1 (3) |
| Mutant (%) | 7 (23) |
| *c.290C>A, p.(Ser97Tyr)* | 5 |
| *c.326A>T, p.(Glu109Val)* | 1 |
| *c.424G>A, p.(Val142Ile)* | 1 |
| **ATTR-targeted therapy** |  |
| Tafamidis (61mg, Vyndamax™) | 17 (47) |
| Tafamidis (20mg, Vyndaqel™) | 1 (3) |
